# The complete chloroplast genome sequence of *Docynia indica* (Wall.) Decne

**DOI:** 10.1080/23802359.2019.1666669

**Published:** 2019-09-18

**Authors:** Dawei Wang, Chen Shi, Hongyan Tang, Chengzhong He, Anan Duan, Hede Gong

**Affiliations:** a Key Laboratory for Forest Resources Conservation and Utilization in the Southwest Mountains of China Ministry of Education, Southwest Forestry University, Kunming, China;; b Key Laboratory for Forest Genetic and Tree Improvement and Propagation in Universities of Yunnan Province, Southwest Forestry University, Kunming, China;; c Puer City Institute of Forestry Sciences, Puer, Yunnan, China;; d School of Geography, Southwest Forestry University, Kunming, Yunnan, China

**Keywords:** *Docynia indica* (Wall.) Decne., chloroplast genome, phylogenetic analysis

## Abstract

*Docynia indica* (Wall.) Decne. (Duo-Yi) is a high economic value for exploitation and utilization wild fruit tree species with edible and medicinal values in southwest China. We have sequenced the chloroplast genome to facilitate genetic improvement of this species and to assess phylogenetic relationships among major lineages. The result showed that the total chloroplast genome size of Duo-Yi was 159,546 bp in length, containing a pair of inverted repeats (IRs) of 26,369 bp, which were separated by a large single copy (LSC) and small single copy (SSC) of 87,650 bp and 19,158 bp, respectively. The overall guanine-cytosine (GC) content of the chloroplast genome was 36.6%. There were 125 genes in the chloroplast genome, which including 82 protein-coding genes, 35 transfer RNA genes, and 8 ribosomal RNA genes. Among these genes, there were 14 genes with one intron and 3 genes have two introns. The result of phylogenetic analysis indicated that the Duo-Yi was closely related to the genera of *Malus doumeri.*

*Docynia indica* (Wall.) Decne., also called Duo-Yi in China, is an evergreen wild fruit tree species belonging to the Rosaceae family, which wildly distributed from East Asia to China (Rymbai et al. 2016). Its leaves are rich in polyphenols and flavonoids, extracts from the leaves have several pharmacological actions such as anti-obesity (Nguyen et al. 2011), antifungal (Zhang et al. 2018a), antioxidant (Sharma et al. 2015), which were widely used as a medicine for the healing of empyrosis, fracture and fever by the local ethnic minorities in China (Lin et al. 2003; Zhang et al. 2018b). The fruits contain high levels of biologically active components including anti-oxidants, tannins and flavonoids, which were used as natural remedy to treat digestive problems and infectious diseases (Tiep et al. 2018). Recent studies have shown that the extracts of fruits have hypoglycemic, hypolipidodemic, lipid-lowering and weight-loss effects (Vivek et al. 2017). Generally, Duo-Yi is a high economic value for exploitation and utilization wild fruit tree species with edible and medicinal values in southwest China.

Chloroplast genetic engineering had become a powerful tool for basic research in biogenesis and function of this organelle (Bausher et al. 2006). The complete chloroplast genomes have extensively been used in resolution of phylogenetic relationships, study of DNA barcoding and genome evolution (Leseberg and Duvall 2009; Dong et al. 2012; Zong et al. 2019). Currently, complete chloroplast genomes of several species from the Rosaceae family have been studied and deposited at the GenBank database (Gichira et al. 2017). These studies have greatly boosted our understanding of phylogenetic relationships in Rosaceae. However, the plastome of Duo-Yi has not been reported. In this paper, we reported the complete chloroplast genome sequence of Duo-Yi employing the high-throughput sequencing approaches. The phylogenetic analysis will provide an examination of relationships among several major clades of Rosaceae family.

Leaf samples of Duo-Yi were obtained from the Puer city (Yunnan, China; geospatial coordinates: 100°17′19″E, 22°59′74″N; Altitude: 1694 m). The total genome DNA was isolated using the Ezup plant genomic DNA kit (Sangon Biotech, Shanghai, China), and DNA samples (Duo-Yi 1-5) were stored at the Key Laboratory for Forest Resources Conservation and Utilization in the Southwest Mountains of China Ministry of Education, Southwest Forestry University, Kunming, China. After DNA extraction, a library with the insertion size of 350 bp was constructed using procedures described by Zong et al. There were approximately 7 G raw data generated from the high-throughput sequencing. Then the soft-ware of Get Organelle (Jin et al. 2018) was employed to assemble the complete chloroplast genome. The assembled chloroplast genome sequence was then annotated using the program Geneious R8 (Biomatters Ltd, Auckland, New Zealand) and manually corrected. The chloroplast DNA sequence with complete annation information was deposited at GenBank database under the accession number MN088849.

The total length of Duo-Yi chloroplast genome was determined to be 159,564 bp with the circular quadripartite structure similar to major angiosperms chloroplast genomes. The genome contained a small single-copy (SSC) region of 19,158 bp and a large single-copy (LSC) region of 87,650 bp, separated by two copies of an inverted repeat (IR) of 26,369 bp. The overall guanine-cytosine (GC) content of the chloroplast genome was 36.6%. The genome was structured with 111 unique genes including 82 distinct protein-coding genes, eight distinct rRNA genes and 35 distinct tRNA genes. There were 6 tRNA genes, 4 rRNA genes and 4 CDS duplicated in the IR regions, making a total number of 125 genes. Among these genes, there were 14 genes (atpF, ndhA, ndhB, petB, petD, rpl2 and rpl16; trnA-UGC, trnI-GAU, trnK-UUU, trnL-CAA; trnV-UAC) with one intron and three genes (clp P, rps12 and ycf3) with two introns Figure 1.

**Figure 1.**
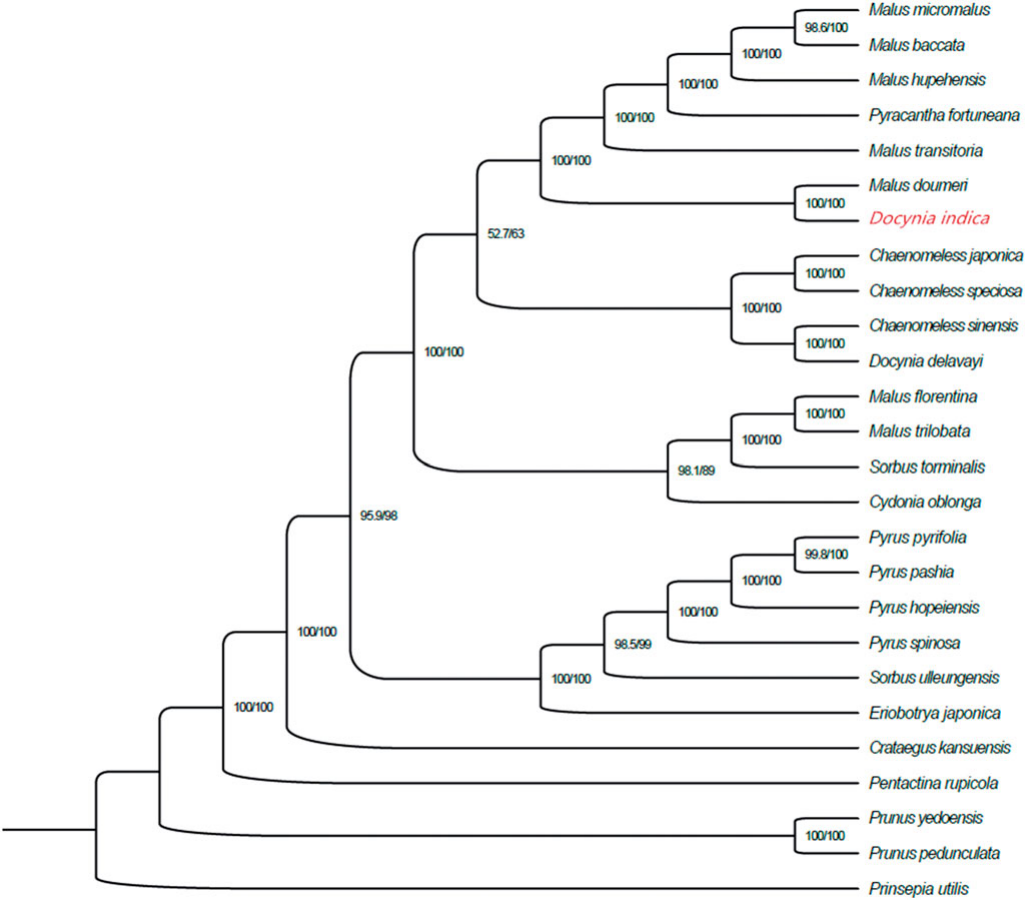
Phylogenetic relationships among 25 complete chloroplas genomes of Rosaceae. Bootstrap support values are given at the nodes. Chloroplast genome accession number used in this phylogeny analysis: *Pentactina rupicola*: JQ041763; *Malus hupehensis*: MK020147; *Malus transitoria*: MK098838; *Malus micromalus*: MF062434; *Malus baccata*: KX499859; *Malus doumeri*: KX499861; *Pyrus hopeiensis*: MF521826; *Eriobotrya japonica*: KT633951; *Malus florentina*: KX499862; *Malus trilobata*: KX499858; *Pyrus pyrifolia*: AP012207.1; *Pyrus pashia*: KY626169; *Pyrus spinosa*: HG737342; *Chaenomeles japonica*: KT932966; *Chaenomeles sinensis*: KT932967; *Chaenomeles speciosa*: KT932965; *Sorbus torminalis*: KY457242; *Pyracantha fortuneana*: MH890570; *Crataegus kansuensis*: MF784433; *Sorbus ulleungensis*: MG011706; *Cydonia oblonga*: KX499857; *Docynia delavayi*: KX499860; *Prunus yedoensis*: KU985054; *Prunus pedunculata*: MG869261; *Prinsepia utilis*: KC571835.

To analyze the Duo-Yi phylogenetic position within Rosaceae lineage, we performed a phylogenetic tree using other 25 chloroplast genome sequences (*P. rupicola*, *M. hupehensis*, *M. transitoria*, *M. micromalus*, *M. baccata*, *M. doumeri*, *P. hopeiensis*, *E. japonica*, *M. florentina*, *M. trilobata*, *P. pyrifolia*, *P. pashia*, *P. spinosa*, *C. japonica*, *C. sinensis*, *C. speciosa*, *S. torminalis*, *P. fortuneana*, *C. kansuensis*, *S. ulleungensis*, *C. oblonga*, *D. delavayi*, *P. yedoensis*, *P. pedunculata*, *P. utilis*) in Rosaceae family by the MAFFT ver-sion 7 software (Katoh and Standley 2013). The maximum likelihood method for phylogenetic analysis was performed by Zong et al. (2019). The neighbour-joining tree showed that the position of Duo-Yi was closely related to the genera of *Malus doumeri (Figure 1).* The result provided vital molecular information to phylogenetic and evolutional study.
